# Preparation of Carboxymethylchitosan Nanoparticles with Acid-Sensitive Bond Based on Solid Dispersion of 10-Hydroxycamptothecin

**DOI:** 10.5402/2011/624704

**Published:** 2011-07-27

**Authors:** Risheng Yao, Lu Liu, Shengsong Deng, Weitao Ren

**Affiliations:** Department of Pharmaceutical Engineering, Hefei University of Technology, Hefei 230009, China

## Abstract

Solid dispersions were prepared by a conventional solvent evaporation method from the water-insoluble model drug 10-hydroxycamptothecin (HCPT) and monomethoxypoly(ethylene glycol) 2000 (mPEG 2000). And then one type of novel biodegradable nanoparticles, the solid dispersion (HCPT/mPEG-CHO) grafted with carboxymethylchitosan (HCPT/mPEG-g-CMCTS) was synthesized. The increase in HCPT solubility of solid dispersion was up to 21-fold compared with the original drug. With the increasing of the amount of mPEG-CHO, solubility of HCPT was from 7.71 **μ**g/mL to 25.82 **μ**g/mL. Colloid systems based on solid dispersion were stable in aqueous medium at 5°C. After 5 months storage at 25°C, the solid dispersions do not change at all. HCPT/mPEG-g-CMCTS was synthesized by grafting reaction of carboxymethylchitosan with mPEG-CHO to form Schiff base which is sensitive to acid environment. The release rate of HCPT from this conjugate in pH 5.4 was much higher than that in the environment of pH 7.4 and p H 4.5. The cumulative release percentages are 45%, 25%, and 15%, respectively. The cumulative release percentage of HCPT in conjugate was only 15% within 85 h while the original drug was up to 70% in pH 7.4, showing a significant slow-release property. This drug model can be attractive candidates as delivery biosystems in tumor therapy.

## 1. Introduction

10-hydroxycamptothecin (HCPT), the natural camptothecin (CPT) analogue, has shown to have a strong antitumor activity against gastric carcinoma, hepatoma, leukemia, and tumor of head and neck in clinical practice [1, 2]. However, the therapeutic potential has been restricted by its low aqueous solubility, *in vitro* and *in vivo* instability. Many recent attempts have been made to improve the shortcomings of HCPT [3–5]. Amongst them is the solid dispersion of the drug into an inert, hydrophilic polymer matrix, which can greatly improve the water solubility of HCPT. Solid dispersions are generally prepared by either a solvent method, whereby the drug and carrier are dissolved in a mutual solvent followed by solvent removal, or by a melting method, whereby drug-carrier mixtures are prepared by comelting/cooling [1, 6, 7]. Another solution is the drug being wrapped into amphiphilic micelles so as to keep stable and release slowly while maintaining their therapeutic efficacy [8–11].

PEGs are widely used as vehicles for solid dispersions because of their rapid solidification rate, capability of forming solid drug solutions, low toxicity, and low costs [12, 13]. Carboxymethylchitosan has been found for the application in biomedical areas because of its biocompatibility and nontoxicity. Besides, due to the presence of reactive amino groups, hydroxyl groups, and carboxyl groups, it can be modified easily to create nano- and microparticles or porous hydrogels which can be employed in a wide range of biomedical applications, such as drug or gene delivery systems [14, 15].

Our long-term aim is to develop a slow-release and acid-activity formulation of HCPT [16–18], which could be useful as attractive candidates in tumor therapy. The present investigation reports a mild method for the preparation of solid dispersions via amino groups grafted with mPEG-CHO. Solid dispersions were prepared by conventional solvent evaporation method and characterized by Fourier transform infrared (FTIR) spectroscopy. We have shown that the interaction between mPEG and HCPT affected drug solubility and stability of drug in the polymer matrix. In this report, we provide evidence that HCPT release from grafted carboxymethylchitosan nanoparticles is significantly slow and sensitive to the tumor environment.

## 2. Methods

### 2.1. Materials

Carboxymethylchitosan (Mw = 360000) was synthesized by ourselves. Poly(ethylene glycol)methyl ether (mPEG, Mn = 2000) was purchased from Sinopharm Chemical Reagent Co. Ltd. Hydroxycamptothecin (HCPT) was purchased from JUNJIE Bio-Technology Co. Ltd. All these materials and other chemicals used in this article were analytic reagent (AR) grade and used as received without further purification.

### 2.2. Synthesis of mPEG-Aldehyde (mPEG-CHO)

mPEG-aldehyde was first prepared by oxidation of mPEG with DMSO/acetic anhydride. After mPEG was completely dissolved into anhydrous DMSO/chloroform solution (90/10, v/v), acetic anhydride was added gradually into the reaction system under the protection of nitrogen atmosphere. The molar ratio of acetic anhydride to mPEG was 12. The reaction system was kept at ambient temperature for 24 h under the nitrogen atmosphere and finally precipitated with excess precold diethyl ether. The mixture was filtered and the obtained crude product of mPEG-aldehyde was reprecipitated twice from chloroform solution with precold diethyl ether. After the drying process, the white mPEG-aldehyde powder was obtained and kept in desiccators for further use.

### 2.3. Preparation of Solid Dispersion (HCPT/mPEG-CHO)

Solid dispersions of HCPT in mPEG-CHO were prepared in different ratios (Table 1) by conventional solvent evaporation method. Briefly, in conventional solvent evaporation method, drug and carrier were dissolved in minimum volume of methanol and chloroform. The reaction mixture was stirred at about 30°C for 24 h; the solvents were removed under vacuum in a rotavapor at 30°C for 0.5 h. The resultant solid dispersion was kept in refrigerator for 2 days to harden. Dispersions were then pulverized in mortar and pestle, and then stored in a desiccator at room temperature. 

### 2.4. Synthesis of Grafted Carboxymethylchitosan Nanoparticles with Solid Dispersion (HCPT/mPEG-g-CMCTS)

**Scheme 1:**
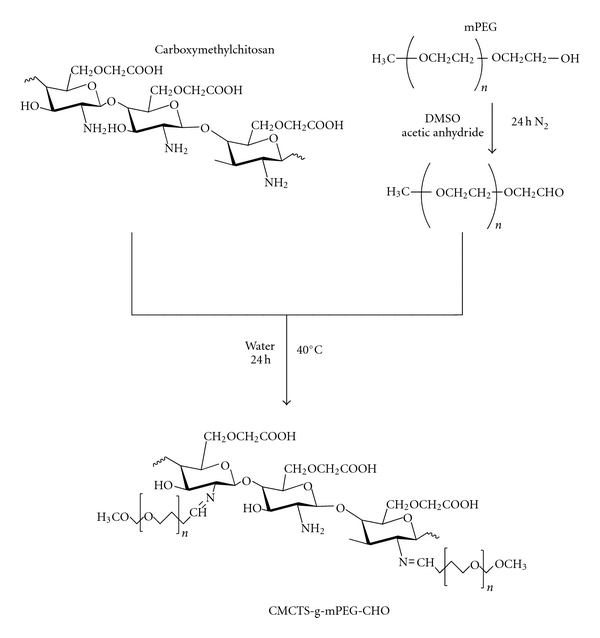
Synthesis scheme of CMCTS-g-mPEG-CHO/HCPT.

HCPT/mPEG-g-CMCTS was synthesized by alkylation of carboxymethylchitosan followed by Schiff base formation as shown in Scheme 1. CMCTS and HCPT/mPEG-CHO (Composition (w/w) of solid dispersions of HCPT is 4/100) were dissolved in 50 mL water separately; CMCTS/water solution was added to the HCPT/mPEG-CHO water solution drop by drop by magnetic stirring. The reaction mixture was stirred at 40°C for 24 h. The solution containing carboxymethylchitosan nanoparticles was purified by dialysis for 3 days against distilled water, then filtered to remove unreacted HCPT and freeze-dried. In the reaction, CMCTS (amino content) and HCPT/mPEG-CHO (aldehyde content) were mixed together at the molar ratio of 1/1.

### 2.5. Characterization of HCPT/mPEG-CHO and HCPT/mPEG-g-CMCTS

#### 2.5.1. Fourier Transform Infrared Spectroscopy (FTIR)

FTIR spectra were performed at room temperature using Nicolet-5700 Infrared Spectrophotometer (USA). The characteristic absorption bands were detected at wavenumbers ranging from 500 to 4500 cm^−1^ using a KBr-pellet method.

#### 2.5.2. ^1^H Nuclear Magnetic Resonance Analysis (NMR)

^1^H NMR spectra were obtained on a Bruker DRX 400 MHz instrument (Switzerland). The samples were dissolved in DMSO. The chemical shifts were represented in ppm, based on the signal for TMS as a reference.

#### 2.5.3. X-Rray Diffraction (XRD)

XRD analysis of dispersions in the form of thin films was performed on randomly oriented samples, scanning over the interval 5–60° 2*θ*, in a Rigaku D/Max2500VC/PC X-ray generator (Japan).

#### 2.5.4. Transmission Electron Microscopy (TEM)

An H-800 transmission electron microscope (Japan) was used to characterize the size and morphology of the dried carboxymethylchitosan nanoparticles. For TEM observation, the solid dispersion dissolved in distilled water and the carboxymethylchitosan nanoparticles were prepared from the reaction mixture after dialysis at a proper concentration. The sample for TEM analysis was obtained by placing a drop of the colloid dispersion containing the chitosan nanoparticles onto a carbon-coated copper grid. It was dried at room temperature and then examined using TEM without any further modification or coating.

### 2.6. Solubility Study of HCPT/mPEG-CHO

An excess amount of HCPT/mPEG-CHO was placed into a vial with 20 mL water. The samples were shaken for at least 6 hours at 25 ± 2°C. 5 mL samples were taken from the saturated solutions and filtered through a 0.22 *μ*m filter. The drug concentration was detected UV spectrophotometrically at *λ* = 384 nm after appropriate dilution with demineralized water and equated to the drug solubility.

### 2.7. Drug Loading Efficiency, Graft Volume, Grafting Degree of Substitution, and Grafting Reaction Rate of HCPT/mPEG-g-CMCTS

To determine the free HCPT during preparation of the HCPT/mPEG-g-CMCTS, 20 mg HCPT/mPEG-g-CMCTS was dissolved in distilled water to a certain concentration. The weight of free HCPT in the solution was determined by UV-vis spectrophotometry (UV-3210, Hitachi, Japan) using a wavelength of 384 nm. The HCPT loading efficiency was calculated as follows:

(i) HCPT loading efficiency (%) = 100 × (the weight of HCPT in HCPT/mPEG-g-CMCTS)/the weight of HCPT/mPEG-g-CMCTS.

The graft volume, grafting degree of substitution and grafting reaction rate which reflect the degree of the reaction were calculated by the product weights as follows:

(i) Grafted volume (%) = 100 × (the quantity of mPEG-CHO in HCPT/mPEG-g-CMCTS)/the quantity of HCPT/mPEG-g-CMCTS.
(ii) Grafting reaction rate (%) = 100 × (the molar volume of mPEG-CHO in HCPT/mPEG-g-CMCTS)/the total molar volume of aldehyde.
(iii) Grafting degree of substitution (%) = 100 × (the molar volume of mPEG-CHO in HCPT/mPEG-g-CMCTS)/the molar volume of carboxymethylchitosan unit.

These data are presented in Table 2.

### 2.8. Release Experiments

All release experiments were performed in a dialysis membrane bag (MWCO = 8,000–16,000) in a shaker incubator (THZ-92A, Boxun, China) at 150 rpm. 20 mL of the micelles with drug solution was placed in the dialysis bag and then immerged in the release medium 300 mL at 37 ± 0.5°C. The phosphate buffer solution (PBS, pH 7.4) was used as the release medium. At the desired time interval, any drug release in the solution was detected by UV spectroscopy (UV-3210, Hitachi, Japan) at 384 nm for HCPT. And then the fresh release medium was added to maintain the constant volume.

Properties of pH-sensitive release experiment carried out in pH = 4.5, 5.4, and 7.4 phosphate buffer solutions.

## 3. Results and Discussion

### 3.1. Preparation of mPEG-CHO

**Figure 1:**
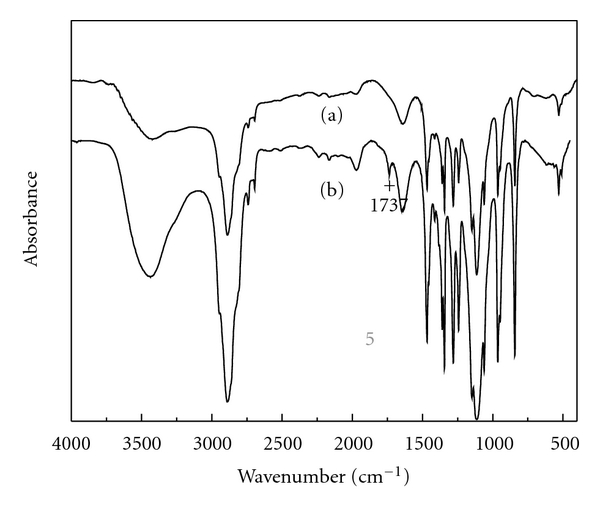
FTIR spectra of HCPT/mPEG-CHO (a), mPEG-CHO (b), and HCPT (c).

It is easy to obtain mPEG-CHO by oxidation of mPEG with DMSO/acetic anhydride.  Figure 1 showed the FTIR spectrum of mPEG-CHO. New formed absorption bands of 1737 cm^−1^ refer to the aldehyde carbonyl C=O stretching vibration, indicating that mPEG-CHO has been successfully synthesized.

### 3.2. Preparation of Solid Dispersion

mPEG-CHO solid dispersion was easy to be produced by the solvent evaporation method. Different weight of HCPT was dissolved in the mPEG-CHO during preparation. Upon cooling, the drug could either remain dissolved in the carrier (solid solution) or precipitate in amorphous or crystalline form (solid dispersion). 

**Figure 2:**
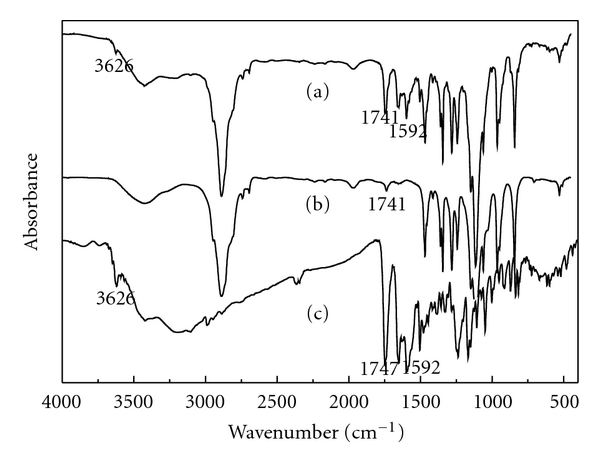
FTIR spectra of mPEG (a) and mPEG-CHO (b).

The FTIR spectrum of HCPT/mPEG-CHO was presented in Figure 2. The spectrum of HCPT/mPEG-CHO is almost the same with mPEG-CHO, which is due to mPEG-CHO occupying most of the proportion of the product. The characteristic absorption bands (3626 cm^−1^ and 1592 cm^−1^) were consistent with HCPT, confirming the presence of HCPT. The aldehyde carbonyl C=O stretching vibration of mPEG-CHO (1741 cm^−1^) merged with the lactone band (1747 cm^−1^) of HCPT, resulting in the peak intensity of the characteristic absorption band (1741 cm^−1^) in HCPT/mPEG-CHO was larger. No new bands formed between HCPT and mPEG-CHO indicated that there is only the physical effect during the preparation process of solid dispersion.

### 3.3. Synthesis of Grafted Carboxymethylchitosan Nanoparticles

Schiff base is a chemical bond which is acid sensitive at pH = 5.4 consistent with the pH of tumor tissue. So that the formed HCPT/mPEG-g-CMCTS particles might give the antitumor drug passive targeting after combining. Therefore, they have great potential for the further use in the drug delivery and release fields. Here we report our successful quest for synthesis of nanoparticles in water without the addition of any surfactant or organic solvent under mild conditions. The grafted Carboxymethylchitosan nanoparticles HCPT/mPEG-g-CMCTS were prepared by the chemical modification of carboxymethylchitosan linear chains using mPEG-CHO at the molar ratio (aldehyde content/amino group content) of 1/1. The product could not only have the advantage of solid dispersion, but also be with the nature of acid activity. 

**Figure 3:**
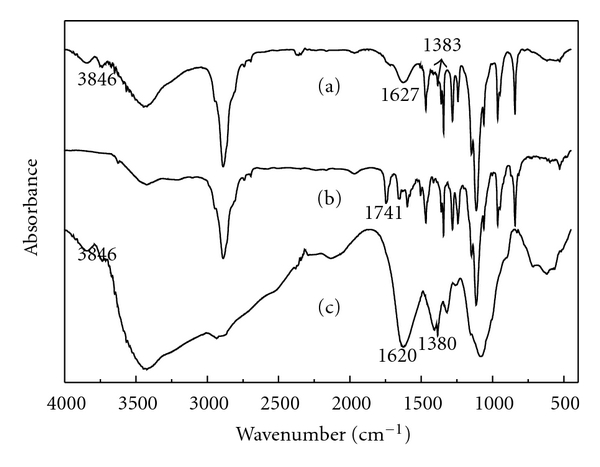
FTIR spectra of CMCTS-g-mPEG-CHO/HCPT (a), HCPT/mPEG-CHO (b), and CMCTS (c).

According to the characteristic spectra of the HCPT/mPEG-CHO, CMCTS, and grafted polymer in Figure 3, an attempt was made to determine the eventual presence of interactions (possible interactions) between carboxymethylchitosan and mPEG-CHO. The characteristic absorption bands (1741 cm^−1^) of HCPT/mPEG-CHO disappeared, indicating that aldehyde has been successfully involved in the reaction. The characteristic absorption bands (3846 cm^−1^ and 1383 cm^−1^) of the grafted polymer were consistent with CMCTS, confirming the presence of CMCTS. The characteristic absorption bands of HCPT/mPEG-g-CMCTS were around 1627 cm^−1^ which confirmed the presence of C=N in polymer. And it (1627 cm^−1^) merged with carboxyl stretching bands (1620 cm^−1^) of carboxymethylchitosan. This illustrates the success of Schiff-base reaction. 

**Figure 4:**
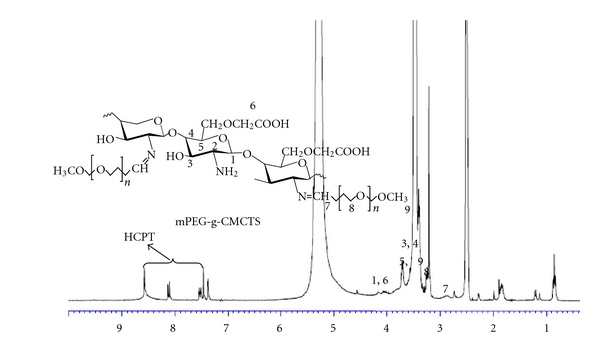
^1^HNMR of CMCTS-g-mPEG-CHO/HCPT.

The structure of grafted polymer was shown in Scheme 1 and the ^1^H-NMR spectrum of grafted polymer was presented in Figure 4, which made further confirmation of its molecular structure. Typical peaks at 3.38–3.71 ppm (H3, 4, 5) and 3.3 ppm (H8) are assigned to the ring methane and methylene protons of carboxymethylchitosan saccharide units and methylene groups of mPEG. The linkage between carboxymethylchitosan and mPEG was confirmed by the appearance of a peak of –N=CHCH_2_O– at 2.59 ppm (H7). Peaks at 7.37–8.57 ppm are attributed to benzene ring protons from HCPT. 

### 3.4. Physical Characterization of Solid Dispersions

**Figure 5:**
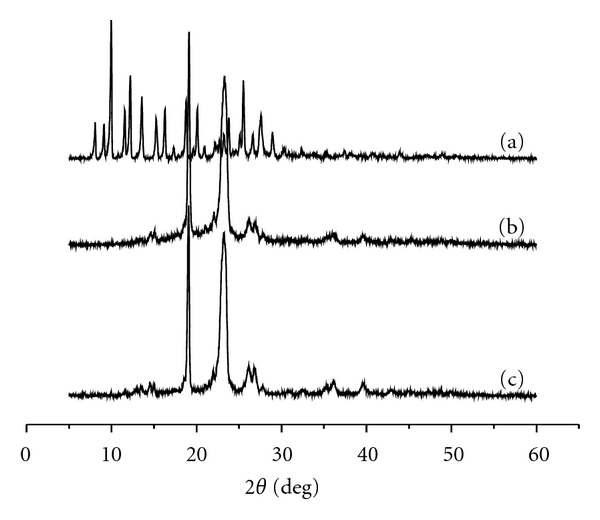
X-ray patterns of HCPT (a), mPEG-CHO (b), and HCPT/mPEG-CHO solid dispersions (c).

XRD diffraction patterns of HCPT, mPEG-CHO, and its HCPT/mPEG-CHO solid dispersions revealed that HCPT is a crystalline compound, showing a very strong diffraction peak at 2*θ* of 10°, 13°, 19°, and 23°. However, even though it is a crystalline material, its dispersions in the mPEG matrix are completely amorphous. Only two broad peaks at 2*θ* of 19° and 23° that correspond to the diffraction pattern of pure mPEG-CHO are recorded, while peaks that correspond to HCPT crystals completely disappear, suggesting that the mPEG-CHO matrix inhibits the crystallization of HCPT (Figure 5).

### 3.5. Solubility Study of HCPT/mPEG-CHO

Table 3 shows the apparent solubility of HCPT of solid dispersions in water at 25°C. MPEG could increase the solubility of HCPT. Compared with the original drug, the increase in HCPT solubility was up to 21-fold. With the increasing of the amount of mPEG-CHO, solubility of HCPT gave out an upward trend which was from 7.71 *μ*g/mL to 25.82 *μ*g/mL. The increase in solubility of HCPT by mPEG-CHO may probably be due to the formation of soluble complexes between water-soluble polymeric carrier and poorly soluble drug. Solubilization of solid dispersion is mainly reflected in two aspects: first is to increase the dispersion of drugs. Drug presented colloidal, microcrystalline, and other highly fragmented state in the carriers with very small particle size which could improve solubility of the drug. Secondly, the carriers can prevent crystallization of the drug. It could keep high-energy state in the carriers so that the solubility was improved. The results indicated that the solubility of HCPT in water could be changed by adjusting the amount of mPEG in solid dispersions, which can meet the different needs in pharmaceutical preparation. 

### 3.6. Morphological Characterization of Solid Dispersions and Carboxymethylchitosan Nanoparticles

**Figure 6:**
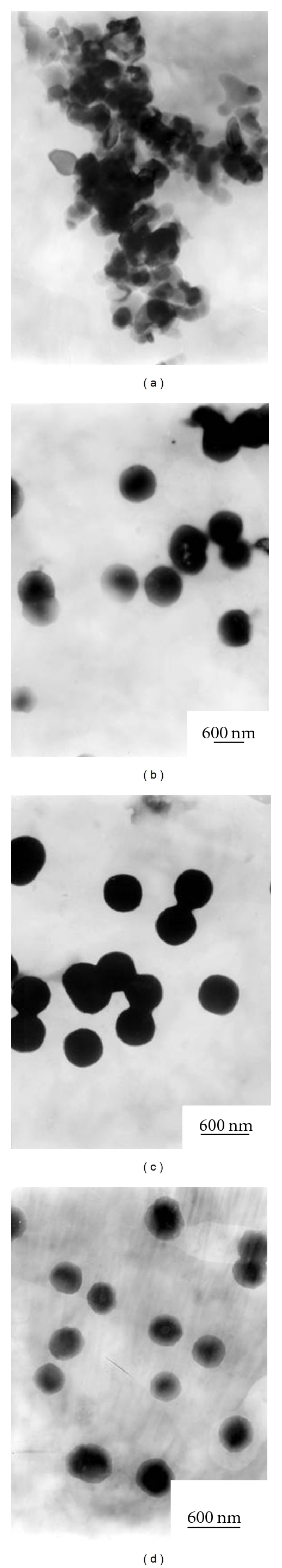
TEM images of solid dispersions after 1 day soaking in water (a), 2 days (b), 7 days (c), and 15 days (d).

Figure 6 presents the TEM images of solid dispersions in water. They could assemble to spherical nanoparticles with regular morphology in aqueous solution. A large number of clusters appear in the view which cannot constitute certain morphology after one day soaking in water; after that, the clusters begin to spread out to form sphere particles, but the aggregation phenomenon still exist. 7 days later, particles become fuller sphere balls with the diameter of 500 nm which are not completely separated. In the following 8 days, all the particles become separate and have good dispersion in water with the diameter of 350 nm. It can be concluded that with the extension of immersing time in water, particles could readjust so that the surface is smoother and the balls are plumper. In addition, the particles have little change after storage for 5 months indicate that the solid dispersions have high drug stability. 

**Figure 7:**
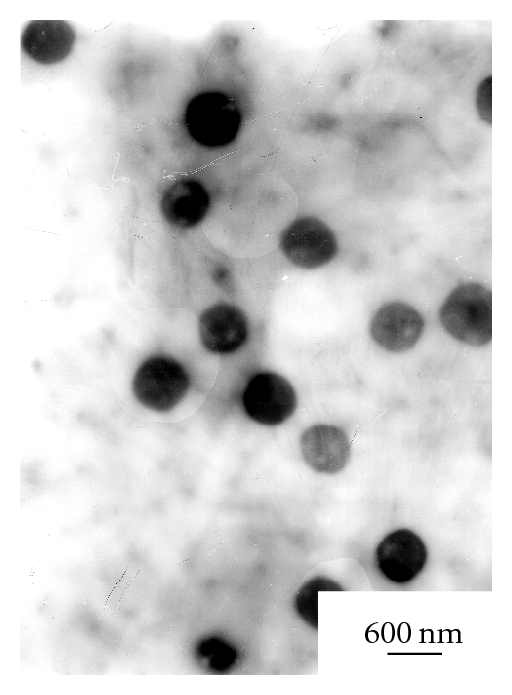
TEM image of HCPT/mPEG-g-CMCTS nanoparticles.

The TEM image of HCPT/mPEG-g-CMCTS nanoparticles was presented in Figure 7. After reacted with carboxymethylchitosan, the diameter of particles went up to 800 nm. But there is little change in particle morphology. All the spherical particles are separate and have good dispersion in water. 

### 3.7. In Vitro Drug Release

#### 3.7.1. Drug Release from Solid Dispersion in Three Different Buffers

**Figure 8:**
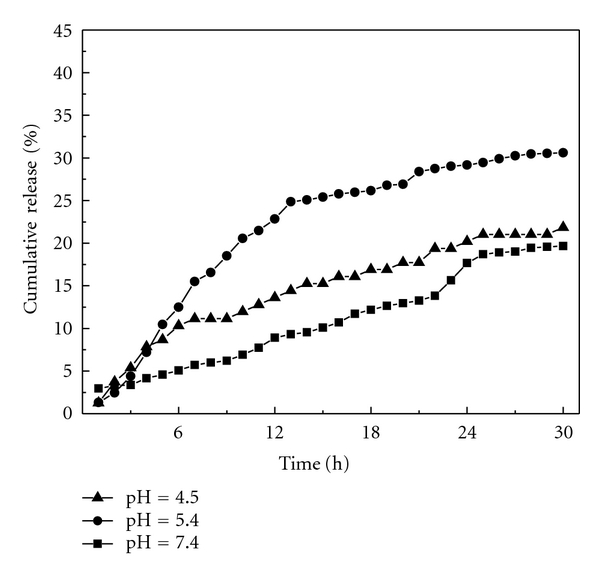
HCPT releasing profiles from solid dispersions in three different buffers.

In an effort to study the effect of release property of solid dispersion on release rates of HCPT from nanoparticles, we have first taken the solid dispersions and compared their release rates in these three buffers. Results were showed in Figure 8. Here, we found that the release rates vary depending upon the release media. The cumulative release percentage are 30%, 20%, and 17% in pH 5.4, pH 4.5, and pH 7.4, respectively. The drug release from solid dispersion was signficantly more in acid environment than that in neutral condition which is the same as our previous work. It should be the inherent nature of mPEG.

#### 3.7.2. Drug Release from Carboxymethylchitosan Nanoparticles in pH 7.4 Buffer

**Figure 9:**
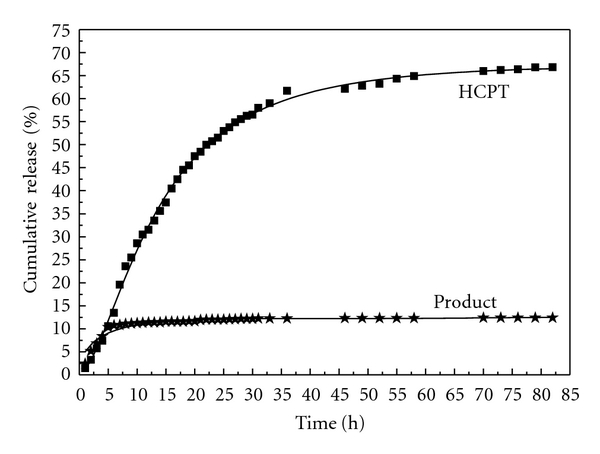
HCPT releasing profiles from nanoparticles in pH = 7.4 buffer.

*In vitro* drug release was performed in pH 7.4 phosphate buffer solution for 85 h. Results of percent drug release versus time for original drug HCPT and HCPT/mPEG-g-CMCTS are presented in Figure 9. The dissolution of pure HCPT occurred very fast, the cumulative release percentage is up to 70% in 85 h. On the other hand, drug-loaded formulations released the drug slowly; it released only about 15% over a period of 85 h. It may be noted that the cumulative release increased slowly after the burst release within 10 h. The reason for this phenomenon was that Schiff base is stable in a relatively neutral condition. Drug release completely accomplished through the diffusion from the drug carrier. In this paper, HCPT was first wrapped in mPEG-CHO and then reacted with CMCTS. So HCPT should first break through the mPEG layer and then the outer CMCTS layer to get into the media. Double force of resistance made the drug release more difficult so that it needed more time to release. 80% of drug release is basically completed in the first 5 h due to the burst release. And higher release rate is observed due to the dissolution of surface-adhered drug from the carrier. At longer time, drug release is due to the diffusion process, which is much slower when compared to the initial release.

#### 3.7.3. Drug Release from Carboxymethylchitosan Nanoparticles in Three Different Buffers

**Figure 10:**
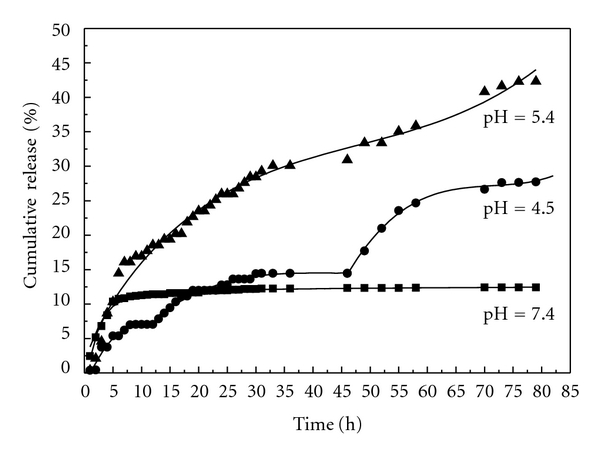
HCPT releasing profiles from nanoparticles in three different buffers.

Figure 10 presents the releasing profiles of HCPT from HCPT/mPEG-g-CMCTS in three different buffers. The release rate of HCPT from this conjugate in pH 5.4 was much higher than that in the environment of pH 7.4 and pH 4.5. The cumulative release percentages are 45%, 25%, and 15%, respectively. Release was rapid at first, and then slowed down, gave out a flat trend at last showing ladder-type model. Such results can be analyzed as follows: in the first 8 h, there was no HCPT in medium so that the concentration gradient is the largest in the whole release process. The dissolution of surface-adhered drug makes the higher release rates. The release curve is similar to straight line; from 8 h to 35 h, the Schiff base bond which is sensitive to pH 5.4 environment cleaved so that the composite structure was broken. Drug in the inner particles began to release and directly contacted with the media. Rapid release rate was observeed again because there was still a large concentration gradient until HCPT reached its temporary equilibrium. The burst release may help maintain the patient therapeutic regime, while the sustained release will maintain the plasma concentration level. 

It was noted that release in pH 7.4 was significantly lower than that in pH 5.4. Most of HCPT released from the particles in the first 10 h because of the Schiff base being stable in a relatively neutral condition. However, we find that HCPT release profile in pH 4.5 is always similar to that in pH 7.4 environment from micelles or hydrogel because Schiff base is stable in such conditions in our previous study. But here the release in pH 4.5 is much different. Release properties of solid dispersion made the release higher in pH 4.5 than in pH 7.4 as shown in Figure 8.

Generally speaking, release of the drug from carboxymethylchitosan nanoparticles involves three different mechanisms: (a) release from the surface of particles, (b) diffusion through the swollen rubbery matrix, and (c) release due to polymer erosion. So we could describe the drug release processes according with kinetics equation *Q* = *k*_1_ *t* ^0.5^ + *k*_2_ *t* + *k*_3_ *t* ^2^ + *k*_4_ *t* ^3^ in the buffer solution. *Q* is the amount of drug released at time *t*, *k*_1_ and *k*_2_ are the rate constants of drug release by diffusion, *k*_3_ is the rate constant of drug release because of diffusion through the swollen rubbery matrix, *k*_4_ is the rate constant of drug release due to polymer erosion. Equations showed as follows:

pH = 7.4: 
  (1) $$\begin{array}{cc} Q & =5.7578t^{0.5}-0.7850t+0.0053t^{2}-2.0000\times 10^{-5}t^{3}\\ R^{2} & =0.9883, \end{array}$$
pH= 5.4:
  (2) $$\begin{array}{cc} Q & =2.9672t^{0.5}+0.8312t-0.0S183t^{2}+0.0001t^{3}\\ R^{2} & =0.9959, \end{array}$$
pH = 4.5:
  - 1–12 h:
    (3) $$\begin{array}{cc} Q & =-3.1571t^{0.5}+3.3726t-0.2171t^{2}+0.0051t^{3}\\ R^{2} & =0.9635, \end{array}$$
  - 13–46 h:
    (4) $$\begin{array}{cc} Q & =-2.4055t^{0.5}+1.7190t-0.0351t^{2}+0.0003t^{3}\\ R^{2} & =0.9668, \end{array}$$
  - 47–82 h:
    (5) $$\begin{array}{cc} Q & =-106.3448t^{0.5}+24.7732t-0.2379t^{2}+0.0010t^{3}\\ R^{2} & =0.9935. \end{array}$$

It can be concluded: drug release caused by diffusion remained the dominant form in pH 5.4 environment, followed by the relaxation of carrier. The rate constant of drug release due to polymer erosion is very small compared to the preceding factors, but should not be negligible; drug release caused by diffusion categorically controlled the whole release process in pH 7.4 condition; result in pH 4.5 is a little complex. There are three stages clearly required to fit the model. In the first 12 h, drug release caused by diffusion and the relaxation of carrier was comparative. 13 h later, diffusion began to control the release process. Gradually, it became the dominant force. And this trend is more obvious after 47 h. Such phenomenon occurs because the release media infiltrating into the carrier have reached its limit, that is, the relaxation of carrier has reached its saturation and does not change any more. Diffusion gradually became the dominant force. Similarly, polymer erosion cannot be ignored.

## 4. Conclusions

We have shown that a nanosized particle assembled from carboxymethylchitosan using a Schiff base reaction with HCPT/mPEG-CHO solid dispersion. The advantage of this drug model lies in two aspects: firstly, solid dispersion can improve the solubility of HCPT greatly in water. In this way, indissoluble drugs could have good dispersion and high wettability in water which can accelerate the rate of drug absorption and improve drug bioavailability; secondly, after reacting with CMCTS, HCPT was more stable *in vivo*, showing a significantly slow release property. In addition, the drug model in our research gave HCPT pH sensitivity which can be attractive candidates as delivery biosystems in tumor therapy.

Compared with the original drug, the increase in HCPT solubility of solid dispersion was up to 21-fold. With the increasing of the amount of mPEG-CHO, solubility of HCPT gave out an upward trend which was from 7.71 *μ*g/mL to 25.82 *μ*g/mL. Colloid systems based on solid dispersion were stable in aqueous medium at room temperature. Particle size of HCPT/mPEG-CHO measured by TEM was around 350 nm which can be achieved for passive targeting of tumor tissue concentration. The drug release processes accorded with kinetics equation *Q* = *k*_1_ *t* ^0.5^ + *k*_2_ *t* + *k*_3_ *t* ^2^ + *k*_4_ *t* ^3^ in the buffer solution. The release rate of HCPT from this conjugate in pH 5.4 was much higher than that in the environment of pH 7.4 and pH 4.5. The cumulative release percentages are 45%, 25%, and 15%, respectively. It meant that the prepared structure of conjugate might be unstable in the acidic environment and the drug would be released sensitively. The cumulative release percentage of HCPT in conjugate was only 15% within 85 h, while the original drug was up to 70% in pH 7.4 environment, showing a significant slow release property.

## Figures and Tables

**Table 1:** Composition (w/w) of solid dispersions of HCPT prepared by conventional solvent evaporation method.

Formulation	HCPT	mPEG-CHO
HCPT/mPEG-CHO (1)	1	100
HCPT/mPEG-CHO (2)	2	100
HCPT/mPEG-CHO (3)	3	100
HCPT/mPEG-CHO (4)	4	100
HCPT/mPEG-CHO (5)	5	100

**Table 2:** Results of drug loading efficiency, graft volume, grafting degree of substitution, and grafting reaction rate of CMCTS-g-mPEG-CHO/HCPT from the equations displayed in Section 2.7.

	Drug loading efficiency (%)	Graft volume (%)	Grafting degree of substitution (%)	Grafting reaction rate (%)
CMCTS-g-mPEG-CHO/HCPT	2.14	75.26	43.55	96.30

**Table 3:** Solubility of solid dispersions.

Composition (w/w) (%)	Absorbance (A)	Solubility (*μ*g/mL)
HCPT	0.093	1.19
1%	0.508	25.82
3%	0.33	16.45
5%	0.164	7.71
